# A Novelty Temperature Compensation Model for Dual-Mass Vibration MEMS Gyroscope Based on Machine Learning and TTAO-VMD Algorithm

**DOI:** 10.3390/mi17010120

**Published:** 2026-01-16

**Authors:** Wenbo Tan, Yan Wang, Xinwang Wang

**Affiliations:** 1 School of Software, North University of China, Taiyuan 030051, China; sz202313020@st.nuc.edu.cn; 2 Sichuan Wizmems Technology Co., Ltd., Chengdu 611731, China; wy@wizmems.com; 3 School of Integrated Circuits, Wuxi University of Technology, Wuxi 214121, China; 4 School of Instrumentation and Engineering, Southeast University, Nanjing 210096, China

**Keywords:** dual-mass vibration, MEMS gyroscope, machine learning, TTAO-VMD, temperature compensation

## Abstract

The output of MEMS gyroscopes is highly vulnerable to ambient temperature variations, which induce temperature drift errors and degrade navigation precision. Consequently, temperature compensation for MEMS gyroscope outputs is of critical importance. To address this issue, this study proposes a novel temperature compensation model for the dual-mass vibration MEMS gyroscope (DMVMG), which integrates the TTAO-VMD, 1D-CNN-Bi-GRU-Attention, and SHAKF algorithms. The implementation process of the proposed model is as follows: firstly, the structural configuration and fundamental operating principle of the DMVMG are elaborated. Secondly, the temperature error compensation model is constructed based on the fusion of the TTAO-VMD, 1D-CNN-Bi-GRU-Attention, and SHAKF algorithms. Thirdly, the raw output signal of the DMVMG is preprocessed using the TTAO-VMD algorithm, which decomposes the signal into four distinct components, namely high-frequency noise, white noise, mixed noise, and temperature-induced noise. Subsequently, the high-frequency and white noise components are eliminated, while the mixed noise component is filtered via the SHAKF algorithm. On this basis, the 1D-CNN-Bi-GRU-Attention algorithm is adopted to establish the temperature error compensation model, with the temperature, temperature change rate, time, and temperature-induced noise as input variables. Finally, the optimized signal components are reconstructed to yield the temperature-compensated output of the DMVMG. The experimental results based on the Allan variance method demonstrate that the angle random walk (N) is reduced from 18.56 ${}^{\circ}/\sqrt{h}$ to 0.17 ${}^{\circ}/\sqrt{h}$, and the bias instability (B) is decreased from 32.76 °/h to 0.82 °/h, verifying the effectiveness of the proposed method.

## 1. Introduction

Micro-Electro-Mechanical System (MEMS) gyroscopes are microscale mechanical gyroscopes featuring low cost, compact size, and high reliability. Unlike traditional gyroscopes, MEMS gyroscopes operate based on the Coriolis effect—a core principle that distinguishes their working mechanism. Benefiting from their inherent advantages of miniaturization, excellent performance, and cost-effectiveness, MEMS gyroscopes have been widely applied in numerous fields, including micro-inertial navigation systems, military equipment, automotive electronics, consumer electronics, mobile devices, robotics, industrial automation, and medical instruments [1]. The material characteristics of the MEMS gyroscope significantly impact its data output accuracy, particularly in terms of temperature, which can cause the output accuracy to fall short of the application’s requirements, thereby limiting its further application [2].

To solve this problem, the MEMS gyroscope is required for temperature compensation. There are two existing temperature compensation methods: device design compensation [3,4,5,6] and algorithm compensation [7,8,9]. Device design compensation is achieved through the design of the internal circuit structure, which prevents changes in the temperature characteristics of the MEMS gyroscope’s internal circuit device due to temperature fluctuations. Algorithm-based compensation refers to a mathematical modeling approach that establishes the correlation between gyroscope output and temperature variations, and then mitigates temperature-induced errors via dedicated algorithms [10,11,12,13]. In contrast to device-oriented compensation methods, the algorithm-based compensation scheme eliminates the need for a sophisticated operating environment and specialized circuit design. Instead, it only requires the collection of adequate raw data to construct an appropriate temperature compensation model, which is capable of predicting the output response of MEMS gyroscopes to temperature fluctuations. This model can achieve high-precision error compensation, thereby effectively enhancing the overall performance of MEMS gyroscopes [14,15].

The temperature compensation model is mainly divided into three methods: traditional filter, neural network, and improved fusion algorithm. Traditional filter temperature compensation models such as the Kalman filter (KF) [14], Extended Kalman filter (EKF), Intrinsic time-scale decomposition (ITD), Wavelet threshold denoising (WTD) [16], and parabolic tracking time–frequency peak filtering (PTTFPF) [17] can have an outstanding performance at denoising the output of the gyroscope. Still, it is not easy to adapt to the nonlinear output caused by complex temperature environments. Ma [18] proposed a novel algorithm, which was ISPO-VMD-BP, that can effectively solve the problem of temperature errors. Cao [11] introduced a processing algorithm that integrates Permutation entropy (PE), Local Characteristic-scale Decomposition (LCD), and an Adaptive network-based fuzzy inference system (ANFIS). The results show that the method effectively eliminates the error and compensates for the drift. Compared to some traditional neural network algorithms, an increasing number of fusion algorithms are being used to establish error compensation models, such as EMD-GA-RBF NN [19], MOVMD-TFPF-BAS-Elman NN [20], and VMD-WLMP-CS-SVR [21]. These fusion algorithms can not only classify and process noise based on the different output frequency but can also more accurately extract the temperature error and significantly improve the accuracy at different temperatures. Meanwhile, machine learning algorithms—including extreme learning machine (ELM) [22], random forest (RF) [23], support vector machine (SVM) [24], logistic regression [25], and K-nearest neighbors (KNN) [26]—have emerged as a crucial research focus and have been extensively applied across a wide range of fields.

To address the performance limitations of the dual-mass vibration MEMS gyroscope (DMVMG) under temperature variations, this paper devises a dedicated denoising method and a temperature error model. A novel temperature compensation model for the DMVMG is also put forward, which combines the advantages of TTAO-VMD (Triangulation Topology Aggregation Optimizer–Variational Mode Decomposition), 1D-CNN-Bi-GRU-Attention (1D-Convolutional Neural Network–bidirectional gated recurrent unit-Attention), and SHAKF (Sage–Husa adaptive Kalman filter). The subsequent content of this paper is arranged as follows: Section 2 outlines the structural characteristics of the target MEMS gyroscope; Section 3 expounds on the proposed fusion method; Section 4 presents the implementation of the temperature experiments, the results of data processing, and a comprehensive comparison of multiple algorithms; and Section 5 concludes the paper with a summary of the major findings and future research directions.

## 2. Dual-Mass Vibration MEMS Gyroscope

### 2.1. The Structure of Dual-Mass Vibration MEMS Gyroscope

**Figure 1:**
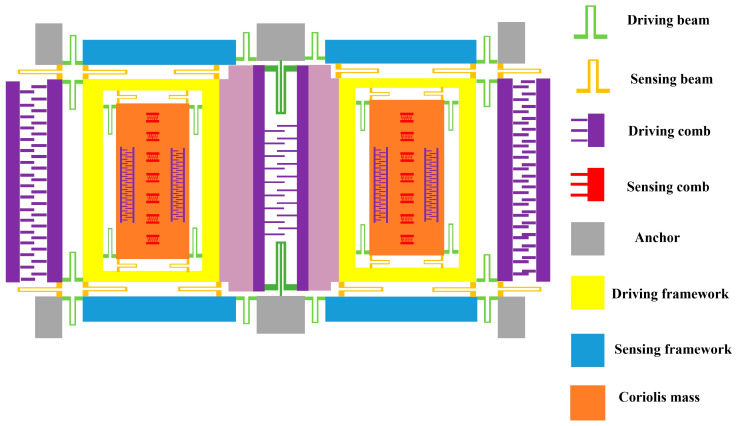
The structure of the DMVMG.

The structure of the DMVMG is shown in Figure 1, which consists of a driving comb, driving framework, sensing comb, sensing framework, driving beam, sensing beam, anchor, and Coriolis mass. The symmetrical distribution of each part forms the left and right gyroscope structure, which are connected by U-shaped beams.

**Figure 2:**
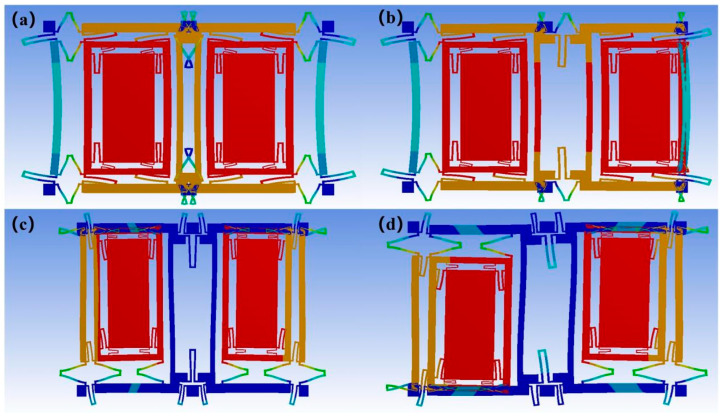
Simulation diagram of the four modes of the DMVMG: (**a**) the driving mode; (**b**) the in-phase mode; (**c**) the in-phase mode; (**d**) the sensing mode.

The modal simulation of the DMVMG was calculated in the software Ansys18.0. In Figure 2a, the first mode is the driving mode, and the two mass blocks perform opposite and same frequency line vibrations in the driving direction, defined as the reverse mode of the mass blocks in the driving direction. The second mode illustrated in Figure 2d corresponds to the sensing mode, where two mass blocks undergo opposite and same frequency line vibrations in the sensing direction, defined as the reverse mode of the mass blocks in the sensing direction. The third mode illustrated in Figure 2b corresponds to the in-phase mode of the mass block along the sensing direction. The fourth mode illustrated in Figure 2c corresponds to the in-phase mode of the mass block along the driving direction. Based on the simulation results and actual processing conditions, the size parameters of the DMVMG are shown in Table 1.

### 2.2. Working Principle of Dual-Mass Vibration MEMS Gyroscope

**Figure 3:**
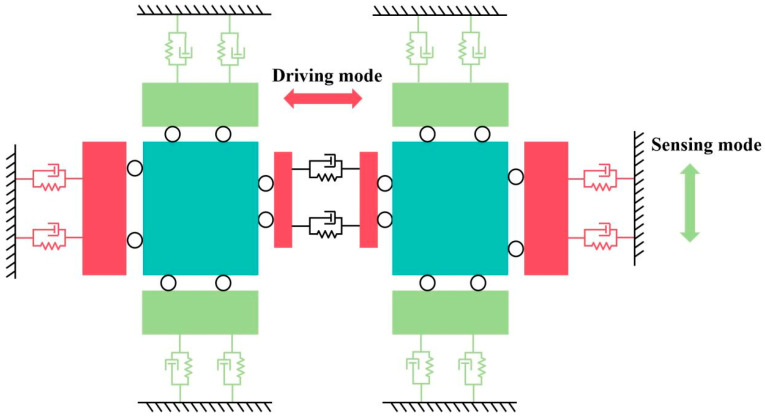
Working principle of the DMVMG.

The working principle of the DMVMG is shown in Figure 3 and the second-order dynamic equation of the DMVMG is as follows:

(1) $$m_{x}x^{\prime \prime }+d_{x}x^{\prime }+k_{x}x=F_{d}\sin(w_{d}t)$$

(2) $$m_{y}y^{\prime \prime }+d_{y}y^{\prime }+k_{y}y=F_{d}\sin(w_{d}t)$$

where *m_x_* represents the driving equivalent mass, *d_x_* represents the driving equivalent damping, *k_x_* represents the driving equivalent stiffness, *m_y_* represents the sensitive equivalent mass, *d_y_* represents the sensitive equivalent damping, and *k_y_* represents the sensitive equivalent stiffness.

### 2.3. Measurement and Control Circuit System

**Figure 4:**
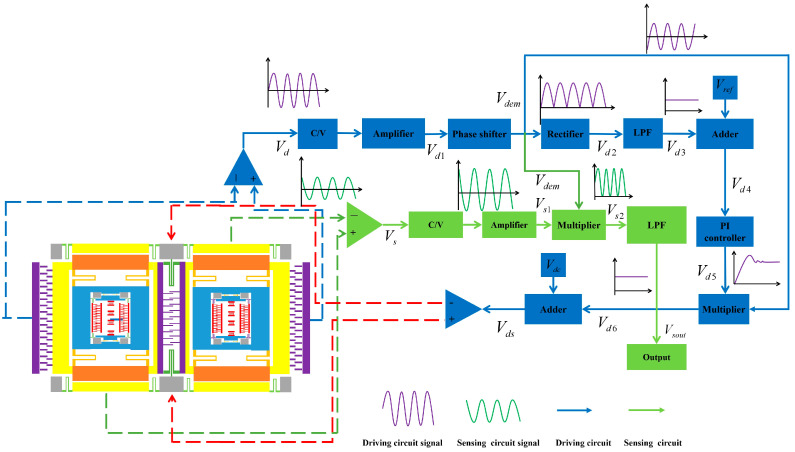
Measurement and control circuit system of the DMVMG.

To keep the DMVMG in normal working condition, the control circuit adopts the method of a driving closed loop (blue module in Figure 4) and a sensing open loop (green module in Figure 4), where the driving loop adopts the closed-loop control method of an AGC-PLL (Automatic Gain Control-Phase Locked Loop). Based on the driving closed loop, the driving signal $V_{d1}$ is first produced by the driving comb and amplified by a differential amplifier. Secondly, the signal is delayed by a phase shifter circuit to match the drive AC signal $V_{dem}$. Thirdly, the signal is envelope-detected by a full-wave rectifier and fourth-order low-pass filter in order to enter a voltage comparator to compare the output with the reference signal $V_{ref}$. The output signal $V_{d4}$ is transferred to the PI controller, which can generate a control signal $V_{d5}$. The signal $V_{ds}$ is modulated by the modulating signal $V_{dem}$ and then superimposed with $V_{dc}$ into the driving closed loop. Based on the sensing open loop, the sensing signal of the Coriolis central disk motion becomes the signal $V_{s1}$ after passing through the C/V converter and the differential amplifier. $V_{s1}$ is demodulated by $V_{dem}$ to produce $V_{s2}$. Finally, $V_{s2}$ is passed through the fourth-order low-pass filter to produce an output open-loop signal $V_{sout}$.

## 3. Algorithms and Models

### 3.1. TTAO-VMD

The traditional VMD algorithm is sensitive to noise, which may lead to signal mode mixing and affect the signal decomposition effect. At the same time, the decomposition layer K and penalty factor α in the VMD algorithm need to be manually selected, and the selection of parameters has a significant impact on the decomposition effect. Therefore, optimizing the VMD algorithm through the Triangulation Topology Aggregation Optimizer can improve its stability and reliability in the MEMS gyroscope output signal decomposition.

**Figure 5:**
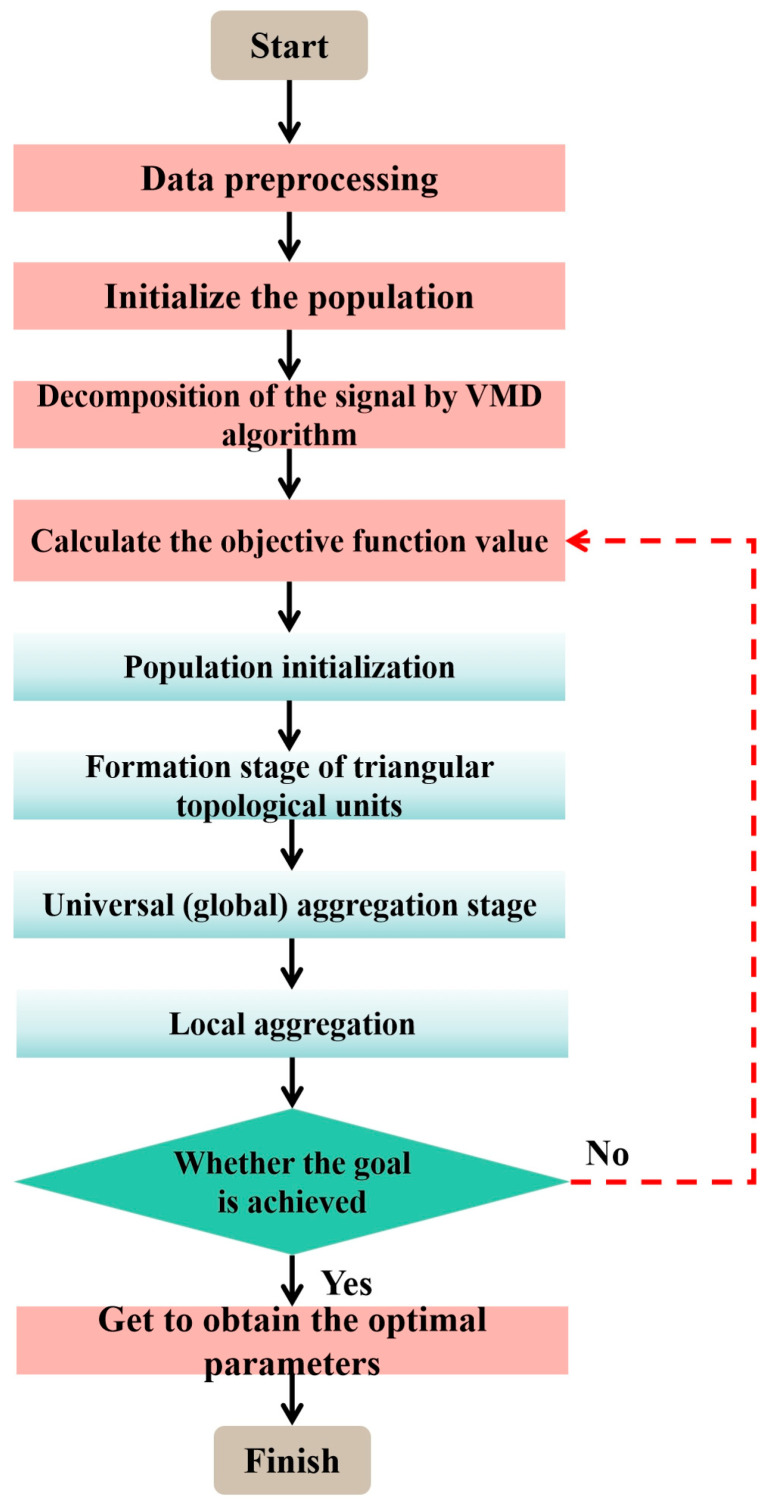
The process of the TTAO-VMD algorithm.

The TTAO algorithm is rooted in the similar triangle principle, adopting an aggregation strategy to gather vertex individuals with valuable information, which can be achieved either between distinct topological units or within a single topological unit. The workflow of the TTAO-VMD algorithm is presented in Figure 5.

(1) Population initialization

First and foremost, the TTAO algorithm initiates the optimization process by initializing the population. Within the TTAO algorithm, the total number of individuals N can be partitioned into N/3 triangular topological units, where [∙] denotes the floor function. Any remaining individuals are randomly generated within the predefined search space. Specifically, in the initialization phase, N/3 agents are randomly sampled from the feasible region, and the mathematical expression governing the generation of each individual is given as follows:

(3) $$\vec{X_{i,1}}=r_{0}\times (\vec{UB}-\vec{LB})+\vec{LB}$$

where $\vec{X_{i,1}}$ represents the first search individual in the *i*-th triangular topological unit, and *i* is a positive integer between 1 and N/3. $r_{0}$ represents random numbers between 0 and 1, and $\vec{UB}$ and $\vec{LB}$ are the lower and upper bounds of the variables.

(2) Formation stage of triangular topological units

A new directional vector of magnitude 1⋅f is constructed in the spherical coordinate system, with the first vertex designated as its initial point. This vector is then converted to the spherical coordinate system via trigonometric transformations, from which the coordinates of the second vertex are derived. Subsequently, a directional vector of magnitude 1⋅f (generated in the preceding process) is rotated counterclockwise by an angle of π/3; the resulting vector undergoes another round of coordinate system conversion to yield the third vertex. The mathematical expressions for the coordinates of these three vertices are given as follows:

(4) $$\vec{X_{i,2}}=\vec{X_{i,1}}+l\cdot \vec{f(\theta )}$$

(5) $$\vec{X_{i,3}}=\vec{X_{i,1}}+l\cdot \vec{f(\vec{\theta +\frac{\pi }{3}})}$$

where the size of the triangular topological unit l is represented mathematically as follows:

(6) $$l=9\cdot e^{-\frac{t}{T}}$$

where *t* represents the current iteration count, *T* represents the maximum number of iterations, and l decreases as the number of iterations increases. f(θ) and $f(\theta +\frac{\pi }{3})$ represent the direction vectors of the other two edges guided by the first point:

(7) $$\vec{f(\vec{\theta })}=[\cos\theta_{1},\cos\theta_{2},\ldots ,\cos\theta_{D-1},\cos\theta_{D}]$$

(8) $$\vec{f(\vec{\theta +\frac{\pi }{3}})}=[\cos(\theta_{1}+\frac{\pi }{3}),\cos(\theta_{2}+\frac{\pi }{3}),\ldots ,\cos(\theta_{D-1}+\frac{\pi }{3}),\cos(\theta_{D}+\frac{\pi }{3})]$$

where $\theta =[\theta_{1},\ldots \theta_{D}]$ and $\theta_{j}(j=1,\ldots ,D)$ are random numbers between 0 and π. For each set of triangular topological units, an internal aggregation operation is performed to generate a fourth vertex, which is constructed via a linear weighting method that incorporates the individual characteristic information of the triangular units, as expressed below:

(9) $$\vec{X_{i,4}}=r_{1}\cdot \vec{X_{i,1}}+r_{2}\cdot \vec{X_{i,2}}+r_{3}\cdot \vec{X_{i,3}}$$

where $r_{1}$, $r_{2}$, and $r_{3}$ are them, and are random numbers between [0, 1] and $r_{1}+r_{2}+r_{3}=1$. Therefore, the fourth search agent is located within each triangular topological unit.

(3) Universal (global) aggregation stage

During this stage, information about elite individuals from different triangular topological units is collected to generate new feasible solutions. Specifically, information interaction is conducted between the optimal individual of each triangular topological unit and the optimal individual selected randomly from an arbitrarily chosen unit set. For each dimensional variable of these two superior individuals, a linear combination with distinct weight coefficients is performed. New individuals are generated through the effective connection of these two “vertex” individuals, and the generation mechanism is mathematically expressed as follows:

(10) $$\vec{X_{i,new1}^{t+1}}=r_{4}\cdot \vec{X_{i,new1}^{t+1}}+(1-r_{4})\cdot \vec{X_{rand,best}^{t}}$$

where $r_{4}$ is a random number between 0 and 1. $X_{i,best}^{t}$ and $X_{rand,best}^{t}$ represent the optimal individual of unit i and the randomly selected unit at the i-th iteration. In addition, adopting a greedy strategy to update the optimal agent, $X_{i,best}^{t}$ and $X_{rand,best}^{t}$ can be updated to the following:

(11) $$\left\{\begin{array}{cc} \vec{X_{i,best}^{t+1}}=\vec{X_{i,new1}^{t+1}} & f_{\vec{X_{i,new1}^{t+1}}}<f_{\vec{X_{i,best}^{t}}}\\ \vec{X_{i,sbest}^{t+1}}=\vec{X_{i,new1}^{t+1}} & f_{\vec{X_{i,new1}^{t+1}}}<f_{\vec{X_{i,sbest}^{t}}} \end{array}\right.$$

where $X_{i,sbest}^{t}$ represents the suboptimal individual at the i-th iteration.

(4) Local aggregation

At this stage, local aggregation is carried out among triangular topological units. Building on the results of the previous stage, a temporary triangular topology is constructed between the updated optimal or suboptimal individual and two vertices with superior fitness values in the group, with the note that this topology does not necessarily take the form of an equilateral triangle. On the basis of the motion vector difference derived from the optimal and suboptimal individuals, the position of the optimal individual is subjected to local disturbance in both direction and step size. As a result, each group performs a re-exploration within a specific local region, thereby enabling the full utilization of each topological triangular element. The position of the new vertex can be calculated according to the following formula:

(12) $$\vec{X_{i,new2}^{t+1}}=\vec{X_{i,best}^{t+1}}+a\cdot (\vec{X_{i,best}^{t+1}}-\vec{X_{i,sbest}^{t+1}})$$

α can be calculated as follows:

(13) $$\alpha =\ln(\frac{e-e^{3}}{T-1}t+e^{3}-\frac{e-e^{3}}{T-1})$$

The incorporation of suboptimal individual information serves to avoid the optimal individual being trapped in local optima. Specifically, if the newly generated individual outperforms the original one in terms of fitness, the position of the original individual will be updated; otherwise, the update operation will not be executed. The corresponding mathematical formulation is presented as follows:

(14) $$\vec{X_{i,best}^{t+1}}=\left\{\begin{array}{cc} \vec{X_{i,new2}^{t+1}} & f_{\vec{X_{i,new2}^{t+1}}}<f_{\vec{X_{i,best}^{t+1}}}\\ \vec{X_{i,best}^{t+1}} & otherwise \end{array}\right.$$

### 3.2. 1DCNN-Bi-GRU-Attention Algorithm

The 1DCNN employs a small set of parameters to capture spatial features of input data, enabling it to improve model accuracy while reducing training time. However, the 1DCNN lacks information storage capability, which tends to result in data loss. In the practical task of predicting temperature errors of MEMS gyroscopes, solely considering the correlation between unidirectional MEMS gyroscope output data and temperature fails to fully capture the dynamic characteristics of the internal temperature in the temperature-controlled chamber as well as its rate of change. To address this limitation and enhance the accuracy of MEMS gyroscope temperature error prediction, the bidirectional gated recurrent unit (Bi-GRU) can leverage the past time-axis information of the temperature-controlled chamber to extract the influences of both the temperature and its change rate on the future output data of the MEMS gyroscope. To further boost the prediction accuracy, the attention mechanism is introduced to assign distinct weights to the contributions of different data points to the MEMS gyroscope temperature error model simultaneously.

#### 3.2.1. Bi-GRU Algorithm

**Figure 6:**
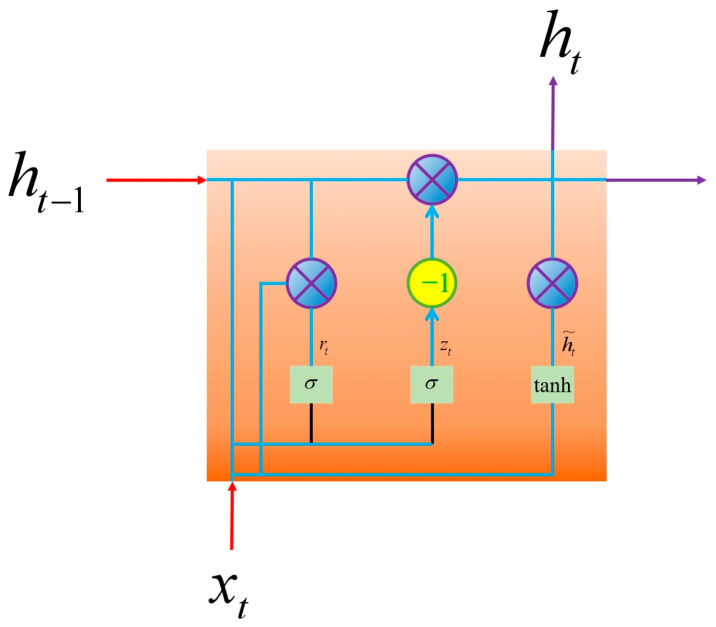
The structure of the GRU.

As a variant of recurrent neural networks (RNNs), the gated recurrent unit (GRU) addresses the issues of information forgetting and gradient vanishing/exploding that plague traditional RNNs when processing long-sequence data by introducing a gating mechanism. The core components of the GRU are the update gate and reset gate, which enable the network to dynamically determine the timing of hidden state updates and resets. Specifically, the update gate regulates the proportion of information to be incorporated into the current hidden state, while the reset gate controls the degree of integration between the current input and the previous hidden state. When the reset gate value approaches 0, the network effectively disregards the information from the previous hidden state; when the update gate value approaches 1, the network prioritizes extracting and retaining information from the previous hidden state. Unlike some other RNN variants, the GRU utilizes a single hidden state for information storage without a separate memory unit. This structural simplicity allows the GRU to achieve more efficient training and temperature error prediction for MEMS gyroscopes, particularly in scenarios with relatively limited datasets. The structure of the GRU is shown in Figure 6. And $z_{t}$ represents the update gate, $r_{t}$ represents the reset gate, $x_{t}$ represents the current input, ${\tilde{h}}_{t}$ represents the summary of the input and past hidden layer states, and $h_{t}$ represents the output of the hidden layer.

**Figure 7:**
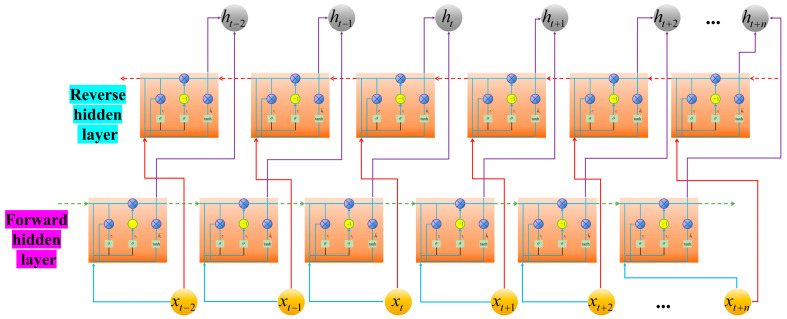
The structure of the Bi-GRU algorithm.

In traditional recurrent neural networks, information data is often transmitted unidirectionally from one moment to another. Nevertheless, in some cases, the current output is governed not only by the state of the prior time instant but also by the information data associated with the subsequent time instant, where both factors hold equivalent significance for the model’s performance. In this case, traditional recurrent neural networks are not sufficient for data mining. Therefore, according to the principle of the Bi-LSTM algorithm, the same method can be applied to the GRU, which consists of one layer of a forward GRU and one layer of a backward GRU. Unlike traditional networks, the current output is influenced by both past and future information data. The structure of the Bi-GRU is shown in Figure 7.

In Figure 7, each current output is determined by three parts: the previous hidden layer output state $h_{t-1}$ propagated forward along the timeline, the previous hidden layer output state $h_{t-1}$ propagated backward along the timeline, and the input $h_{t-1}$ at the current time.

The combination process of each output $y_{t}$ can be represented by Equations ((15)–(17)):

(15) $$h_{t}=GRU(x_{t},h_{t-1})$$

(16) $$h_{i}=GRU(x_{t},h_{i-1})$$

(17) $$y_{t}=w_{t}h_{t}+w_{i}h_{i}+b_{t}$$

where GRU() represents the computational process of traditional GRU networks; $h_{t}$ represents the forward hidden layer state; $h_{i}$ represents the backward hidden layer state; $y_{t}=w_{t}h_{t}+w_{i}h_{i}+b_{t}$ represents the output weight matrix for the hidden layer of the forward propagation unit; $w_{t}$ represents the output weight matrix for the hidden layer of the backward propagation unit; and $b_{t}$ represents the hidden layer bias vector for the current moment.

#### 3.2.2. Attention Mechanism Algorithm

The attention mechanism is a way to achieve network adaptation, which assigns different weights to different information to ignore inefficient or ineffective information and focus attention on practical details. Although the Bi GRU structure can mine the temperature information of MEMS gyroscope output data from the bidirectional time dimension, not all temperature information of MEMS gyroscope output data is practical, which may lead to information overload. Therefore, an attention mechanism is needed to compensate for this drawback.

Given that the output data of MEMS gyroscopes incorporates time-stamped information, error characteristics corresponding to different time intervals exert distinct impacts on angular velocity measurements. For instance, the initial error within a specified time window accumulates progressively over time, which in turn induces notable error deviations at the end of the time window. However, the bidirectional gated recurrent unit Bi-GRU algorithm exhibits inherent limitations in capturing and predicting the time segment-specific variations in the MEMS gyroscope output data. This paper introduces an attention mechanism to differentiate the relative importance of data segments across different time intervals. The mathematical expression of the attention mechanism is specifically given as follows:

(18) $$a_{i}=\frac{\exp(score_{i})}{\sum_{i=1}^{t-1}\exp(score_{i})}$$

where $a_{i}$ represents the importance of the *i*-th time window for predicting the output of the MEMS gyroscope, and $score_{i}$ is the weight of attention.

#### 3.2.3. 1D-CNN-Bi-GRU-Attention Model

**Figure 8:**
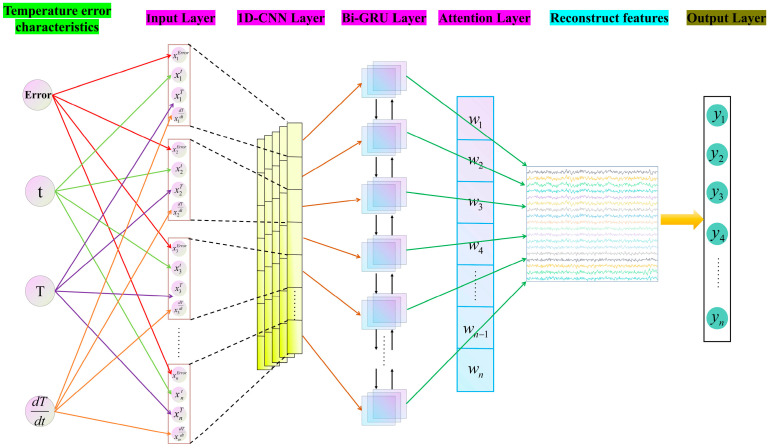
The structure of the 1D-CNN-Bi-GRU-Attention model.

The 1D-CNN-Bi-GRU network model constructed in this article consists of five layers, which are the MEMS gyroscope temperature error information input layer, 1D-CNN layer, Bi-GRU layer, attention layer, and output layer. The temperature error information of the MEMS gyroscope is extracted through the 1D-CNN for feature extraction, and then the Bi-GRU and attention layer are used to learn the temporal information rules for better prediction performance. Finally, the output prediction result is obtained. The neural network structure is shown in Figure 8. The steps of the 1D-CNN-Bi-GRU-Attention model are below.

Step 1: Preprocess the temperature error of the MEMS gyroscope.

Step 2: Based on the characteristics of the temperature error output of the MEMS gyroscope in the one-dimensional timing signal, feature extraction is achieved through the 1D-CNN network. Firstly, the 1D-CNN network structure alternates between the convolutional layer and the pooling layer for data processing. Then, expand the high-dimensional nodes of the data and activate it through the ReLU function. Finally, the fully connected layer is used for structural transformation to extract feature vectors and output them.

Step 3: Perform temporal analysis on the feature information output by the 1D-CNN layer to learn temporal patterns. A Bi-GRU network is built to extract features from the output information of the upper neural network and analyze the patterns of information changes.

Step 4: The weight matrix of the temperature error information of the MEMS gyroscope is obtained by continuously iterating the feature vectors obtained in step 3 based on Formulas (15)–(17). The formula for calculating the weight coefficients is as follows:

(19) $$e_{t}=u\tanh(wh_{t}+b)$$

(20) $$a_{t}=\frac{\exp(e_{t})}{\sum_{i=1}^{t}e_{i}}$$

(21) $$s_{t}=\sum_{t=1}^{i}a_{t}h_{t}$$

where $h_{t}$ is the output vector of the Bi-GRU layer at time t, $e_{t}$ is the corresponding probability distribution value of attention, u and w are the weight, b is the bias, and $s_{t}$ is the attention layer output at time t.

Step 5: After the temperature error of the MEMS gyroscope is processed by the attention layer, the output is obtained through a fully connected Dense network. The output information with a step size of l can be represented as $Y=[y_{1},y_{2}\ldots y_{l}]$. The activation function adopted in the fully connected layer is the Sigmoid function, and the calculation formula for the information processing of the fully connected layer is as follows:

(22) $$y_{t}=Sigmoid(w_{0}s_{t}+b_{0})$$

where the output $s_{t}$ is $y_{t}$, $w_{0}$ and $b_{0}$ are, respectively, weights and biases; the activation function is the Sigmoid function.

The Mean Squared Error (MSE) function is used as the loss function, which is as follows:

(23) $$MSE=\frac{1}{n}\sum_{i=1}^{n}{\left(y_{i}-{\hat{y}}_{i}\right)}^{2}$$

where n is the number of samples, i is the index of the observed values, $y_{i}$ is the actual value of the i-th observation value, and ${\hat{y}}_{i}$ is the predicted value of the *i*-th observation.

### 3.3. SHAKF Algorithm

Due to the uncertainty of noise statistical characteristics of MEMS gyroscopes under whole temperature conditions and the possibility of interference from external factors such as the environment and temperature, the estimation accuracy is reduced, which seriously affects the reliability and real-time performance of the filter. In practical applications, noise is random and chaotic. There is no doubt that using traditional methods of fixed filtering parameters can lead to filter divergence. A Sage–Husa adaptive filter is an adaptive filtering algorithm designed to address the unpredictable statistical characteristics of noise. Adding a time-varying noise estimator to the traditional Kalman filter framework enables the real-time estimation and correction of noise statistical characteristics, which effectively suppressed the occurrence of filter divergence. Therefore, the SHAKF is used to denoise the output data of the MEMS gyroscope based on the Kalman filter in this article. By introducing the forgetting factor and increasing the weight index of unfamiliar parameters, the real-time estimation and adjustment of process noise and observation noise in MEMS gyroscopes are carried out based on filtering residuals, which can reduce the impact of external factors such as the environment and temperature on the output data prediction of the MEMS gyroscope.

For general linear systems, the discretized state equation can be expressed as follows:

(24) $$X_{k}=A_{k-1}X_{k-1}+W_{k-1}$$

(25) $$Z_{k}=H_{k}X_{k}+V_{k}$$

where $X_{k}$ represents the state variable, $Z_{k}$ represents the measurement vector, $H_{k}$ represents the measurement matrix, $A_{k}$ represents the state matrix, $W_{k-1}$ represents the process noise, and $V_{k}$ represents the measurement noise. The traditional Kalman filter algorithm is divided into a prediction process, correction process, and updating error covariance:

Prediction process:

(26) $${\hat{X}}_{k/k-1}=A_{k-1}{\hat{X}}_{k-1}+B_{k-1}u_{k-1}$$

(27) $$P_{k/k-1}=A_{k-1}P_{k-1}A_{k-1}^{T}+\Gamma_{k-1}Q_{k-1}\Gamma_{k-1}^{T}$$

Correction process:

(28) $$K_{k}=P_{k/k-1}H_{k}^{T}{\left(H_{k}P_{k/k-1}H_{k}^{T}+R_{k}\right)}^{-1}$$

(29) $${\hat{X}}_{k}={\hat{X}}_{k/k-1}+K_{k}(Z_{k}-H_{k}{\hat{X}}_{k/k-1})$$

Updating error covariance:

(30) $$P_{k}=(I-K_{k}H_{k})P_{k/k-1}$$

where $K_{k}$ is the gain of the Kalman filter, $P_{k/k-1}$ is the prior error covariance, $P_{k}$ is the error covariance, ${\hat{X}}_{k/k-1}$ is the prior estimate, ${\hat{X}}_{k}$ is the posterior estimate, and P(W)~(0,Q),P(V)~(0,R).

Based on the Equations (24) and (25), considering stochastic linear discrete systems, the SHAKF is derived and Equations (24) and (25) can be rewritten as follows:

(31) $$X_{k}=AX_{k-1}+q_{k}+W_{k-1}$$

(32) $$Z_{k}=H_{k}X_{k}+r_{k}+V_{k}$$

Simplifying Formulas (31) and (32) can obtain the following:

(33) $${\hat{X}}_{k/k-1}=A{\hat{X}}_{k-1}+{\hat{q}}_{k-1}$$

(34) $$e_{k}=Z_{k}-H_{k}{\hat{X}}_{k/k-1}-{\hat{r}}_{k}$$

(35) $${\hat{X}}_{k}={\hat{X}}_{k/k-1}+K_{k}e_{k}$$

where ${\hat{q}}_{k-1}$ is the estimated mean of the process noise, ${\hat{r}}_{k}$ is the estimated mean of the measurement noise, $e_{k}$ is the innovation matrix, $K_{k}$ is the Kalman filter gain, ${\hat{X}}_{k}$ is the state estimation vector, and ${\hat{X}}_{k/k-1}$ is the prior estimate of time k at time k−1.

The covariance matrix and gain are as follows:

(36) $$P_{k/k-1}=AP_{k-1}A^{T}+{\hat{Q}}_{k-1}$$

(37) $$K_{k}=P_{k/k-1}H_{k}^{T}{\left(H_{k}P_{k/k-1}H_{k}^{T}+R_{k}\right)}^{-1}$$

(38) $$P_{k}=(I-K_{k}H_{k})P_{k/k-1}$$

In Equations (39)–(42), the specific formulas based on ${\hat{r}}_{k}$, ${\hat{R}}_{k}$, ${\hat{q}}_{k}$, ${\hat{Q}}_{k}$, $\lambda =1-d_{k}$ are as follows:

(39) $${\hat{r}}_{k}=\lambda {\hat{r}}_{k-1}+d_{k}(Z_{k}-H_{k}{\hat{X}}_{k/k-1})$$

(40) $${\hat{R}}_{k}=\lambda {\hat{R}}_{k/k-1}+d_{k}(e_{k}e_{k}^{T}-H_{k}P_{k/k-1}H_{k}^{T})$$

(41) $${\hat{q}}_{k}=\lambda {\hat{q}}_{k-1}+d_{k}({\hat{X}}_{k}-A{\hat{X}}_{k-1})$$

(42) $${\hat{Q}}_{k}=\lambda {\hat{Q}}_{k-1}+d_{k}{\left(K_{k}e_{k}e_{k}^{T}K_{k}^{T}+P_{k}-AP_{k-1}A^{T}\right)}_{k}$$

where $r_{k}$ is the mean of the process noise and $R_{k}$ is the mean of the measurement noise. $q_{k}$ is the covariance matrix of the measurement noise, $Q_{k}$ is the covariance matrix of the process noise, $d_{k}=(1-b)/(1-b^{k+1})$ is the correction factor, $d_{0}=1$, b is the forgetting factor usually ranging from 0.95 to 0.99.

### 3.4. Temperature Compensation Model

**Figure 9:**
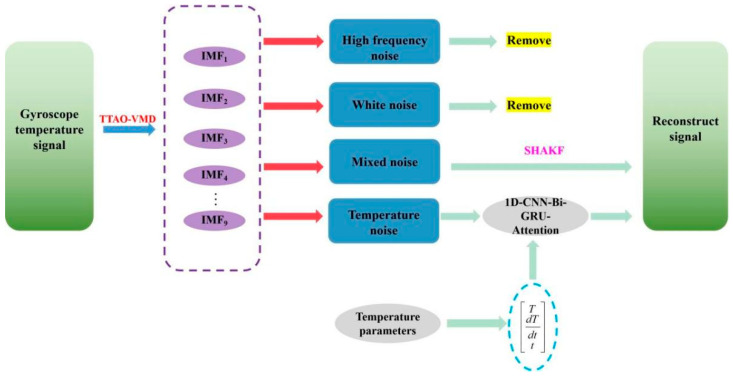
The structure of the temperature compensation model.

A novel temperature compensation model for the DMVMG is proposed, which integrates the TTAO-VMD algorithm, 1D-CNN-Bi-GRU-Attention network, and SHAKF algorithm. The implementation process of this model is detailed as follows:

Firstly, preprocessing is performed on the original output signal of the DMVMG. Specifically, based on the TTAO-VMD algorithm, the original signal is decomposed into four signal components, namely high-frequency noise, white noise, mixed noise, and temperature noise. Secondly, targeted noise elimination is carried out as follows: high-frequency noise and white noise components are directly removed due to their negligible effective information for gyroscope output. Subsequently, the SHAKF algorithm is adopted to denoise the mixed noise component, yielding a denoised signal ①. Next, a temperature noise compensation model is constructed using the 1D-CNN-Bi-GRU-Attention algorithm. In this model, the temperature T, temperature change rate dT, time *t*, and temperature noise are selected as the input variables to establish a temperature error compensation mechanism. Through this model, compensation is performed on the temperature-related noise component, resulting in a compensated temperature signal ②. Finally, signal reconstruction is implemented as follows: the denoised signal ① (obtained after SHAKF-based mixed noise denoising) and the compensated temperature signal ② (obtained after temperature noise compensation) are fused and reconstructed. The signal generated from this reconstruction serves as the final temperature-compensated output signal of the DMVMG. The complete implementation process of the proposed temperature compensation model is illustrated in Figure 9.

## 4. Experiment and Analysis

### 4.1. Experimental Data Collection

**Figure 10:**
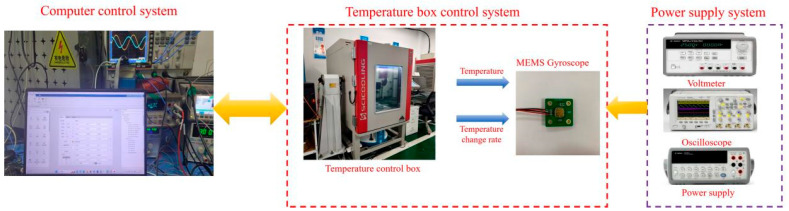
Temperature experimental environment of the DMVMG.

**Figure 11:**
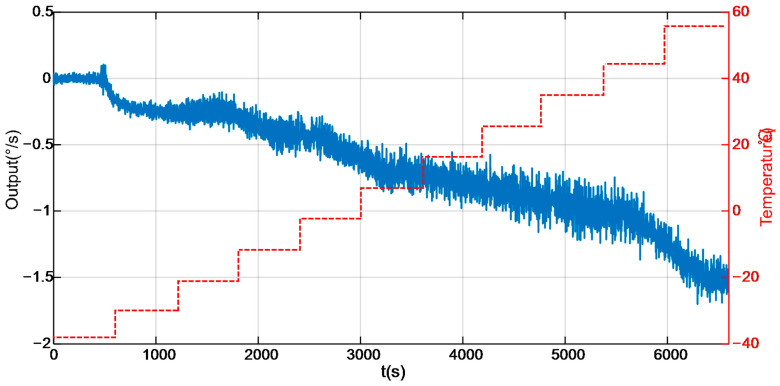
The output of the DMVMG range from −40 °C to 60 °C.

Figure 10 illustrates the experimental setup for the tests, which is primarily composed of a computer control system, a temperature chamber control system, and a power supply system. To characterize the temperature-dependent performance of the DMVMG, the gyroscope was mounted on a temperature-controlled chamber throughout the entire experimental process [27]. The experimental procedure was carried out as follows: Firstly, the initial internal temperature of the chamber was set to −40 °C, and the frequency of the DMVMG was configured to 1 Hz. Secondly, the DMVMG was powered on, and the experiment was initiated once the gyroscope’s output signals stabilized. Subsequently, the temperature rise rate was set to 0.1 °C/min and the internal temperature of the chamber was gradually increased from −40 °C to 60 °C. The output characteristics of the DMVMG over the temperature range of −40 °C to 60 °C are presented in Figure 11 [28].

### 4.2. Analysis of Experimental Results

**Figure 12:**
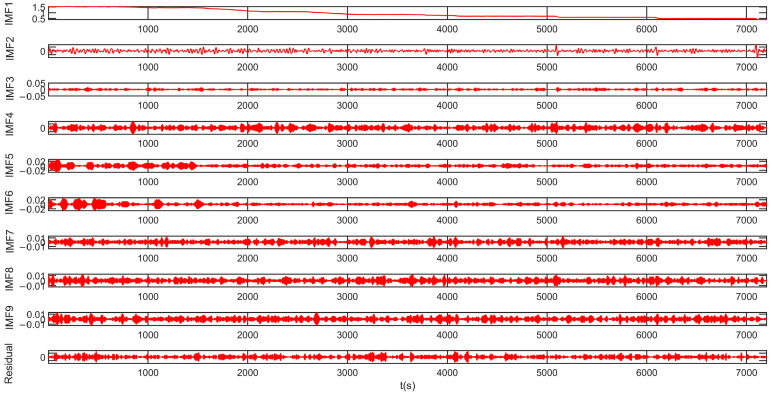
IMF components based on the TTAO-VMD algorithm.

To compensate the output signal of the DMVMG, the TTAO-VMD algorithm is adopted in this study. The key parameters of the TTAO-VMD algorithm consist of the population size, $r_{1}$, $r_{2}$, and $r_{3}$. Specifically, the population size is configured to 60, and the value range of $r_{1}$, $r_{2}$, and $r_{3}$ is set as [0, 1]. Additionally, the optimal number of decomposition modes K is determined to be 9, and the penalty factor α is assigned a value of 35.72. The intrinsic mode functions (IMFs) decomposed by the TTAO-VMD algorithm are presented in Figure 12.

**Figure 13:**
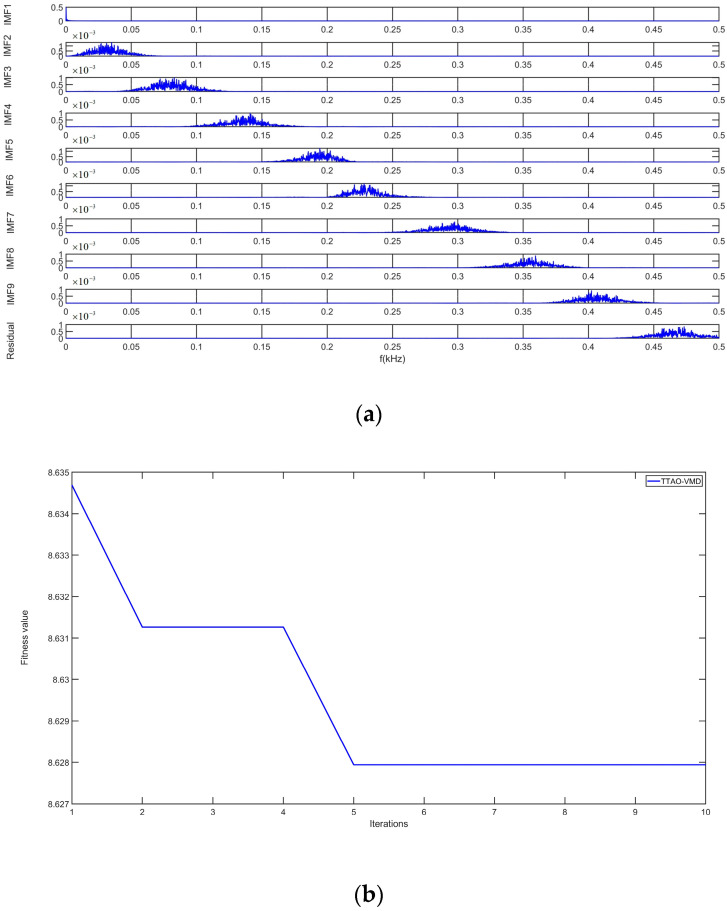
The related parameters curve of the TTAO-VMD algorithm. (**a**) IMFS distribution based on different frequencies; (**b**) The iteration curve of TTAO-VMD algorithm.

In principle, the TTAO-VMD algorithm is capable of effectively decomposing the original signal into nine intrinsic mode functions (IMF1–IMF9) according to the frequency characteristics. Each IMF adaptively corresponds to a fixed bandwidth and center frequency. Through the decomposition process, the TTAO-VMD algorithm can successfully extract the high-frequency noise, white noise, mixed noise, and temperature noise components. Ultimately, nine IMFs are obtained, which can reflect both the temperature drift characteristics and noise information of the original signal, as shown in Figure 13.

The 1D-CNN-Bi-GRU-Attention algorithm is built using the TensorFlow deep learning framework and compiled using Pycharm [29]. Due to the complexity of the 1D-CNN-Bi-GRU-Attention model, the model parameters need selecting, which are usually manually adjusted based on experience. The parameters of the 1D-CNN-Bi-GRU-Attention algorithm are shown in Table 2.

**Figure 14:**
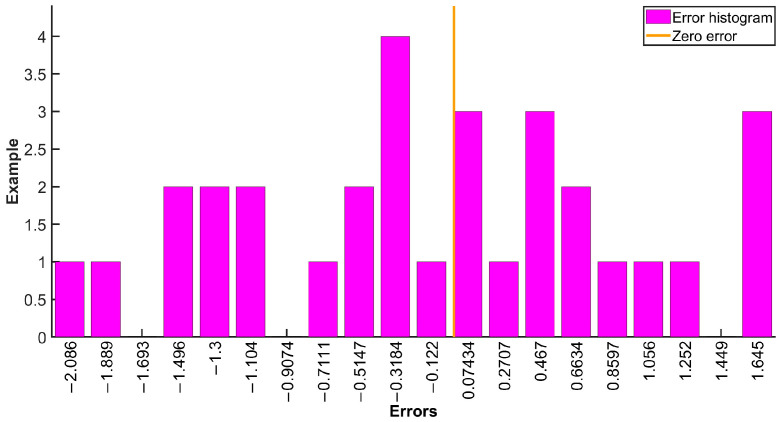
The error histogram of the 1D-CNN-Bi-GRU-Attention algorithm.

**Figure 15:**
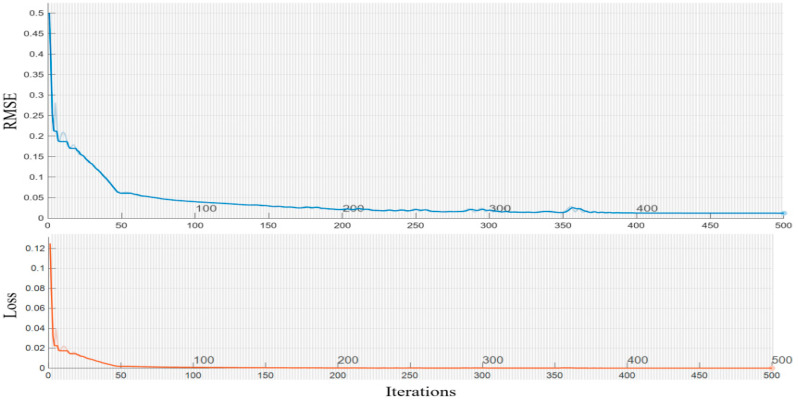
Iterations of the 1D-CNN-Bi-GRU-Attention algorithm.

**Figure 16:**
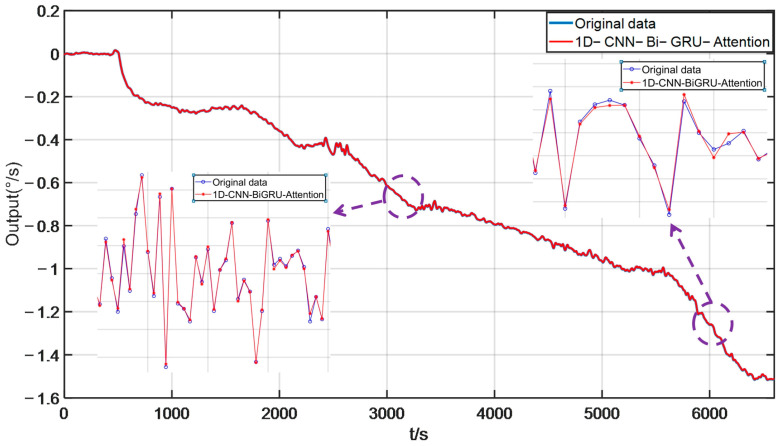
The temperature noise of the DMVMG after temperature error model compensation.

Dropout is a regularization technique used to reduce the degree of overfitting in deep learning models. Epoch is the number of iterations the model undergoes during training. Batch_size represents the number of training samples used to update the model parameters each time. The size of the value affects the convergence speed of the model. Learning rate is a hyperparameter that affects the convergence speed and stability of a model, and the optimizer is set to the Adam algorithm. The error histogram of the 1D-CNN-Bi-GRU-Attention algorithm is displayed in Figure 14. From Figure 14, it can be seen that the error histogram of the 1D-CNN Bi GRU Attention algorithm is reasonable and suitable for the temperature model. Iterations of the 1D-CNN-Bi-GRU-Attention algorithm are shown in Figure 15. From Figure 15, it can be seen that the loss value will continuously decrease and tend to stabilize towards zero. At the same time, according to the loss rate curve, it also indicates that the model training effect is relatively ideal, and the actual output can well approximate the theoretical output. The temperature noise of the DMVMG after temperature error model compensation is demonstrated in Figure 16. From Figure 16, it can be seen that the temperature error model of the DMVMG is basically consistent with the real output error, indicating that the 1D-CNN Bi GRU Attention algorithm can track the temperature error of the DMVMG well [30].

**Figure 17:**
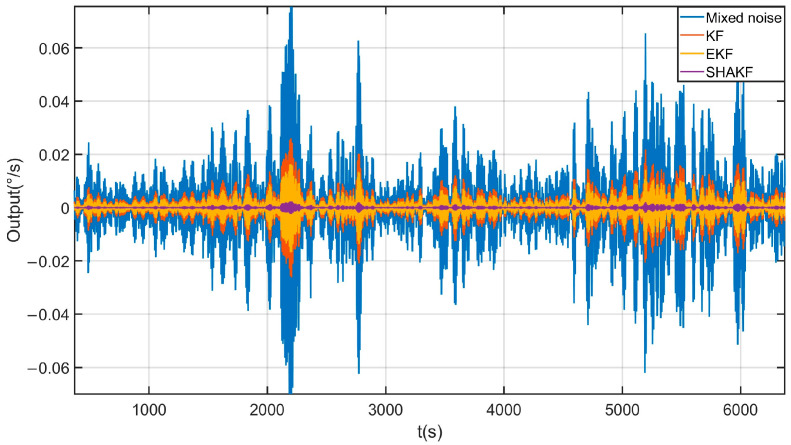
The filtering effects of the KF, EKF, and SHAKF based on mixed noise.

**Figure 18:**
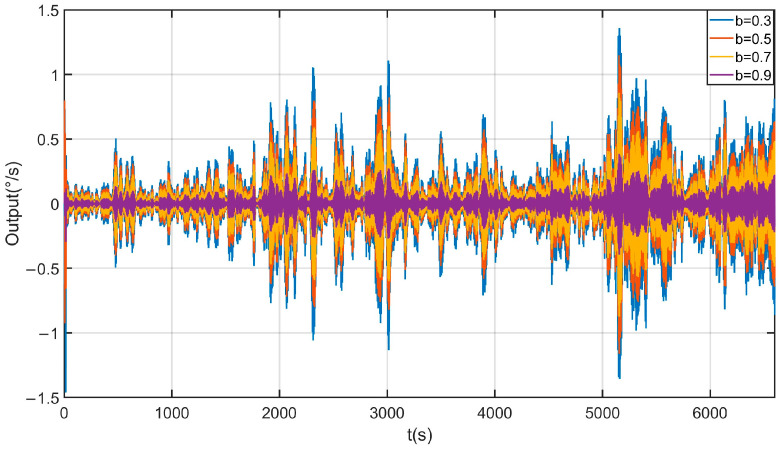
The impact of different values for forgetting factor B on the effectiveness of the SHAKF.

**Figure 19:**
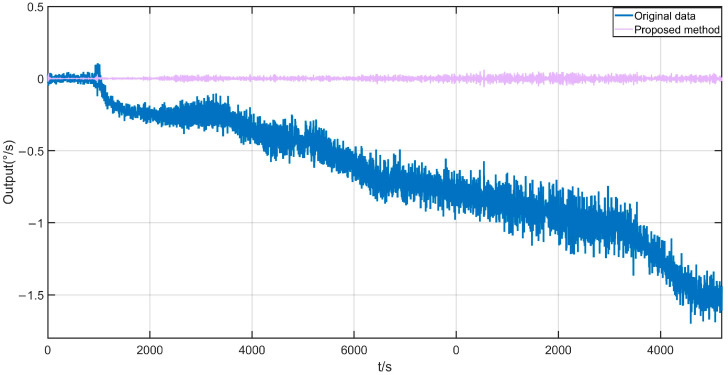
The reconstructed signal of the proposed method.

Due to the introduction of the forgetting factor b in the SHAKF, this article not only discusses the filtering effects of three methods, KF, EKF, and SHAKF, but also discusses the impact of different values of the forgetting factor on the effectiveness of the SHAKF. The filtering effects of the KF, EKF, and SHAKF are shown in Figure 17. From Figure 17, it can be seen that the KF, EKF, and SHAKF can all effectively filter mixed noise, but the SHAKF has the best filtering effect. The impact of different values for forgetting factor B on the effectiveness of the SHAKF is demonstrated in Figure 18. From Figure 18, it can be seen that different parameters B have a certain impact on the filtering effect of the SHAKF, and within a certain range, the larger the value of B, the better the filtering range. After comparing the filtering effects, the forgetting factor b is set as 0.9. The reconstructed signal of the proposed method is shown in Figure 19. From Figure 19, it can be seen that the temperature error of the reconstructed signal is greatly suppressed compared to the original signal, and the error of the reconstructed signal is significantly smaller [31].

### 4.3. Allan Variance Analysis

**Figure 20:**
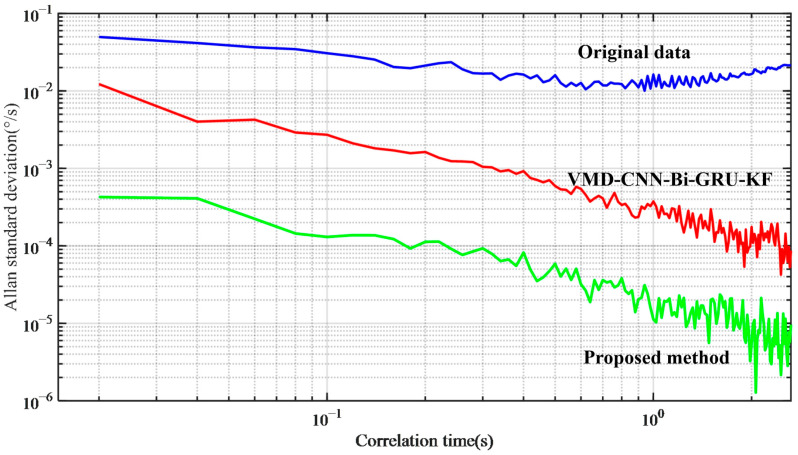
Allan variance before and after compensation.

The Allan variance method is adopted to further process the zero-bias data of MEMS gyroscopes both before and after modeling, which is dedicated to analyzing the various error parameters of MEMS gyroscopes. The Allan variance curves obtained before and after modeling are presented in Figure 20. Table 3 provides a comparison of parameters B and N processed by the proposed method with those obtained via other methods. Compared with the original data, the method proposed in this paper has a significant effect. Among them, B decreased from 32.76 °/h to 0.82 °/h and N decreased from 18.56 ${}^{\circ}/\sqrt{h}$ to 0.17 ${}^{\circ}/\sqrt{h}$. The experimental results show that the method proposed in this paper can significantly improve the output accuracy of the MEMS gyroscope under different temperature environments.

## 5. Conclusions

This paper proposes a novel temperature compensation model for the DMVMG, which integrates three key technologies: TTAO-VMD, 1D-CNN-Bi-GRU-Attention, and SHAKF. To acquire the temperature-dependent output data of the DMVMG, a comprehensive temperature experiment was conducted first. Subsequently, the TTAO-VMD algorithm was employed to decompose the collected temperature output data, and different types of decomposition errors were processed separately using the 1D-CNN-Bi-GRU-Attention and SHAKF algorithms, thereby establishing the proposed temperature error compensation model. Finally, the processed signals were reconstructed to generate the optimized output signal of the DMVMG. The experimental results demonstrate the high accuracy and strong practicality of the proposed method. Based on the Allan variance analysis, compared with the uncompensated output data, the angle random walk (N) is reduced significantly from 18.56 ${}^{\circ}/\sqrt{h}$ to 0.17 ${}^{\circ}/\sqrt{h}$, and the bias instability (B) is decreased remarkably from 32.76 °/h to 0.82 °/h. These results fully validate the superior performance of the proposed temperature compensation model in improving the output precision of the DMVMG.

## Figures and Tables

**Table 1:** The parameters of the DMVMG.

Structure Parameters	Value
The thickness of structure (µm)	30
The mass of driving mode (kg)	1.454 × 10^−7^
The damping coefficient of driving mode (N/(m/s))	1.355 × 10^−6^
The displacement of driving mode (µm)	3.2
The mass of sensing mode (kg)	6.920 × 10^−8^
The damping coefficient of sensing mode (N/(m/s))	8.701 × 10^−5^
The displacement of sensing mode (µm)	0.036
The first mode (Hz)	9375.3
The second mode (Hz)	6852.9
The third mode (Hz)	9236.7
The fourth mode (Hz)	9361.2
The thickness of structure (µm)	30

**Table 2:** The parameters of 1D-CNN Bi GRU Attention algorithm.

Network Structure	Value
Number of first convolution kernels	32
Number of second convolution kernels	64
Dropout	0.3
Optimizer	Adam
Epoch	500
Number of hidden layers in Bi-GRU	2
Learning rate	0.01
Batch_size	128

**Table 3:** Comparison of Allan variances for different methods.

Method	B (°/h)	N (${}^{\circ}/\sqrt{h}$)
Original data	32.76	18.56
Bi-GRU	11.49	5.62
VMD-CNN-Bi-GRU-KF	3.56	2.57
BPTT-LSTM	7.88	3.31
EMD-RBFNN-GA-KF	3.589	-
VMD-SE-WFLP-CSSVR	4	-
Proposed method	0.82	0.17

## Data Availability

Data is contained within the article.
